# Oscillating high-aspect-ratio monolithic silicon nanoneedle array enables efficient delivery of functional bio-macromolecules into living cells

**DOI:** 10.1038/srep15325

**Published:** 2015-10-16

**Authors:** Daisuke Matsumoto, Ramachandra Rao Sathuluri, Yoshio Kato, Yaron R. Silberberg, Ryuzo Kawamura, Futoshi Iwata, Takeshi Kobayashi, Chikashi Nakamura

**Affiliations:** 1 Department of Biotechnology and Life Science, Tokyo University of Agriculture and Technology, 2-24-16 Naka-cho, Koganei, Tokyo, 184-8588, Japan; 2 Biomedical Research Institute, National Institute of Advanced Industrial Science and Technology (AIST), Central5 1-1-1 Higashi, Tsukuba, Ibaraki, 305-8565, Japan; 3 Department of Mechanical Engineering, Shizuoka University, 3-5-1 Johoku, Hamamatsu, Shizuoka, 432-8561, Japan; 4 Research Center for Ubiquitous MEMS and Micro Engineering, AIST, 1-2-1 Namiki, Tsukuba, Ibaraki 305-8564, Japan

## Abstract

Delivery of biomolecules with use of nanostructures has been previously reported. However, both efficient and high-throughput intracellular delivery has proved difficult to achieve. Here, we report a novel material and device for the delivery of biomacromolecules into live cells. We attribute the successful results to the unique features of the system, which include high-aspect-ratio, uniform nanoneedles laid across a 2D array, combined with an oscillatory feature, which together allow rapid, forcible and efficient insertion and protein release into thousands of cells simultaneously.

In the past decade, much progress has been made in controlling cell fate and behavior by using newly developed techniques, including reprogramming of pluripotent stem cells using Yamanaka factors^1^, silencing gene expression by RNAi^2^ and genomic DNA editing using site-specific nucleases^3^. Cells for modeling or for treating of diseases, which are modified using these techniques, have great potential for use in both clinical applications and in biomedical research. In order to achieve the desired cell modifications using the various techniques, it is a crucial first step to efficiently deliver functional bio-macromolecules, DNAs, RNAs or proteins, through the plasma membrane and into the living cell. Conventional methods used for the delivery of materials into live cells often require biological reagents such as plasma membrane-penetrating peptides^4^, virus vectors^5^ or chemical reagents such as lipofectamine^6^ and cationic polymers^7^. The latter, which are widely used for enhancing material transport into the cell, rely on endocytosis and thus often have the disadvantage of low endosomal escape efficiency^8^. That has led to the development of more direct, physical approaches for transferring material into the cell. Techniques such as electroporation or microinjection present alternative solutions, yet suffer from their own drawbacks of low cell viability and low throughput, respectively^9^.

More recently, new studies in cell manipulation have employed devices containing nano-scale acicular materials to allow direct access through the plasma membrane and into the cytoplasm of living cells. A few examples include the measurement of action potential of live cells using nanopillar electrodes^10^, transfer of enzymes^11^ and siRNA^12^ using nanowires, and injection of cobalt ions^13^ and plasmid DNA^14^ using hollow nanostructures. However, penetration of nanostructures through the plasma membrane often proves hard to achieve due to the structural flexibility of the plasma membrane^15^. It was previously reported that successful penetration of a nanoneedle through the cell membrane requires a mechanical force ranging from several nN^16^ to several tens nN^17^. The use of force loading for insertion of multiple nanoneedles, such as a nanoneedle array, into cells, has been investigated previously, and several successful results were reported, including insertion due to material’s own weight^11^ or centrifugation^18^,^19^. However, temporal and spatial control of the contact between the nanoneedles and cells proved to be difficult using these methods. In addition, naked plasmid DNA delivery efficiency using nanoneedle arrays was still very low at around few percent without reagents that inhibit DNA degradation^18^,^19^. In order to facilitate efficient delivery of biomolecules, we undertook to develop a high-aspect-ratio nanoneedle array with an accurate manipulation system, to allow rapid, forcible and efficient insertion and material delivery into live cells.

We have previously reported the development of high-aspect-ratio nanoneedles, which have opened an entirely new avenue for intracellular biochemical and biomechanical analyses of live cells^16^,^20^,^21^,^22^,^23^. Standard silicon pyramidal AFM tips were sharpened, using focused ion beam, into high-aspect-ratio needle-shape structures of 200 nm in diameter. We demonstrated that repeated insertions (>50) of the nanoneedle into a single cell did not affect cell viability^23^. Moreover, we successfully delivered GFP-encoding plasmid DNA into single primary cultured human mesenchymal stem cells, using this direct delivery method, achieving an efficiency of over 70%^20^,^21^. The use of a single nanoneedle enables a simple operation when investigating single cells. However, for therapeutic clinical applications and large-scale analyses, it is necessary to manipulate vast numbers of cells.

In this paper, we demonstrate the development of an array platform of high-aspect-ratio silicon nanoneedles, which allows an efficient, high-throughput delivery of functional biomolecules into thousands of cells simultaneously. One unique aspect of our system is the ability to accurately position the nanoneedle array above the cells and to employ piezoelectric-driven oscillation of the whole nanoneedle array during penetration into the cells. Here, we demonstrate that our nanoneedle array system allows efficient insertion of the nanoneedles and successful delivery of plasmid DNA and Cre proteins into the live cells.

## Results

### Fabrication of nanoneedle array

**Figure 1:**
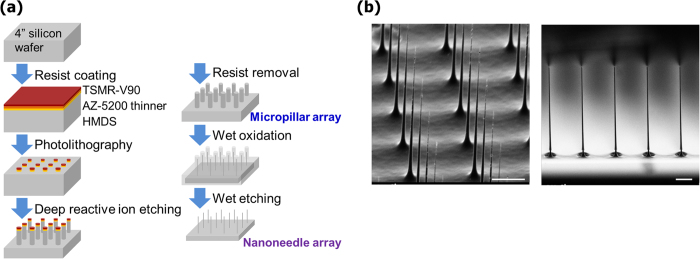
Fabrication of the nanoneedle array. (**a**) Schematic representation showing the steps involved in the fabrication. Micropillars are produced by Deep Reactive Ion Etching (DRIE), followed by thermal wet oxidization and chemical etching steps leading to formation of Si-nanoneedle array. (**b**) Scanning electron microscope images of fabricated nanoneedle arrays. The nanoneedles are spaced uniformly (left) and have high aspect-ratio (right). Scale bars are 5 μm.

We fabricated the nanoneedle arrays from Silicon (Si) wafers using a top-down MEMS approach (Fig. 1a). Array dots (1.8 μm in diameter) of TSMR-V90 (positive photo-resists) were printed using photo-lithographically on a 4-inch Si-wafer with thickness of 400 μm using a stepper (1500MVS, Ultratech). Next, a micropillar array was produced by deep reactive ion etching (DRIE; STS-MUC21, Sumitomo Precision Products), in which it underwent 200 cycles of alternate etching/passivation treatments with SF_6_/C_4_F_8_. Nanoneedles were produced by thinning down the pillars using thermal wet oxidization at 1100 °C (10 h) and buffered hydrogen fluoride (BHF) etching. The nanofabricated Si-wafer was then diced into half-centimeter devices using laser stealth dicing (DFL 7340, Disco) to yield individual devices with 100 × 100 nanoneedles (30 μm pitch), or 300 × 300 nanoneedles (10 μm pitch) in a 3 × 3 mm^2^ area.

A key point in the production process was the DRIE stage. In initial trials, we fixed the etching time by SF_6_ for 2.6 s, which resulted in an overly narrow-bottom shape of the nanoneedles (Supplementary Fig. 1a). This tendency was particularly apparent in the nanoneedles that were made using the wider-pitch photomasks of 30 μm and 50 μm (data not shown). We speculated that micropillars density may have affected convection of the etching gas and that the narrow-bottom shape was attributed to the etching efficiency, especially at latter cycles. Then, we decreased the SF_6_ etching time gradually during each cycle, which resulted in the formation of tapered-shaped nanoneedles with a thicker lower part (Supplementary Fig. 1a). We have then continued to optimize several other parameters, including photo-resist dot size, pitch distance between nanoneedles, and SF_6_ etching time, in order to obtain cylindrical-shaped long micropillars, and adjusted thermal oxidation times to finally achieve the fabrication of cylindrical, high-aspect-ratio nanoneedles.

In the fabrication process, we succeeded in obtaining uniform micropillar arrays, as can be seen in FE-SEM images (Supplementary Fig. 1b **top**). The resulting diameters and lengths of these micropillar arrays were reproducible and similar in each chip produced (Supplementary Table 1). The final step of thermal oxidation yielded nanoneedles that were approximately 200 nm in diameter and over 20 μm in length (Fig. 1b and Supplementary Fig. 1b **bottom**). In addition, the variance in nanoneedles’ length across the chip was very low, with a coefficient of variation (CV) of 5.2% at most, a fact that is crucial for the successful simultaneous insertion of all nanoneedles on the chip into cells cultured on the flat surface (Supplementary Table 2). The variance in nanoneedles’ diameter, however, was relatively high with a CV of 24% at most (Supplementary Table 2). The deviation in diameter might be attributed to the difficulty in controlling the oxidized layer’s thickness on a submicron level. However, that variance in nanoneedles’ thickness wasn’t regarded as a problem, as confirmed by high cell viability following insertion of nano-needles with diameters of up to 400 nm, as shown in a previous research^24^.

### Development of array manipulator

**Figure 2:**
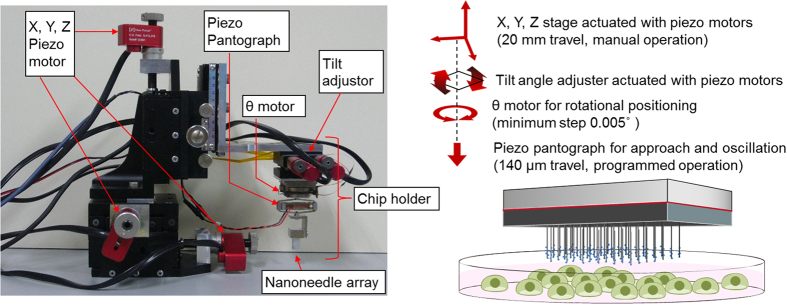
Manipulation of the nanoneedle array. Picture of the manipulator used to control the movement of the nanoneedle array. The movement of the nanoneedle array is controlled by piezoelectric elements, allowing an accurate aligning and positioning of the nanoneedle array on top of the cells. Photograph taken by authors.

Next, we developed an array manipulator, which consists of an XYZ linear translation stage (TBM-605CL, SIGMA KOKI) for coarse adjustments and a chip holder for fine adjustments (Fig. 2). The XYZ stage was actuated with three piezo motors (Model 8321, Newport Corp.), at rates of 0.1~30 μm/s. The chip holder’s XY rotation, tilt angle and Z-axis fine motion were controlled with a θ motor (precision motorized rotation stage) with a resolution of 0.005° (DT70-0062, Technohands), a tilt stage (TD-302, CHUO PRECISION INDUSTRIAL) actuated with two piezo motors (Model 8353, Newport Corp.) and a piezo pantograph (APA120S, Cedrat Technologies), respectively. The piezo pantograph is a unique part of the array manipulator, as it allows the nanoneedle array to be oscillated vertically using a function generator (WF1973, NF corporation) and an actuator driver (ENP-150U, Echo Electronics) at a set frequency and amplitude. The nanoneedle array chip was manually mounted on the chip holder, and all of the following array manipulation was carried out under microscopic observation (Detailed manipulation of nanoneedle array is described in ‘Methods’). Mechanical force of several tens of nN, enough to allow penetration through the cell membrane^16^,^17^, was exerted on each nanoneedle by pressing down the chip until the nanoneedles were in contact with the culture dish and even slightly bent. The monolithic nature of the nanoneedles array enhances its mechanical strength and helps avoiding breaking of the nanoneedles. In addition, oscillation of the nanoneedle array chip using the piezo pantograph contributed to the efficient insertion of the nanoneedles into the cells and also facilitated a quick release of the adsorbed biomolecules from the nanoneedle surface to the cell’s cytoplasm.

### Confirmation of nanoneedles’ insertion into cells

**Figure 3:**
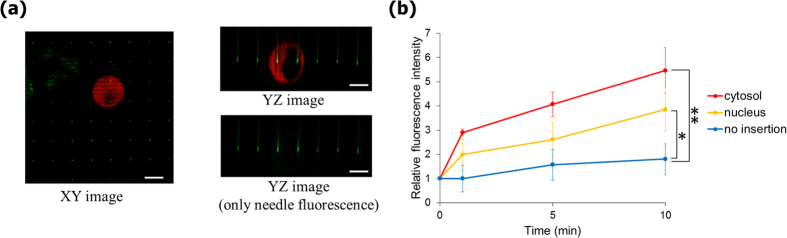
Confirmation of nanoneedles insertion into live cells. (**a**) XY (left) and YZ (right) CLSM images of MB-modified nanoneedle arrays 10 min after insertion into HEK293 cells. (**b**) Time course of the relative fluorescence intensities of nanoneedles following insertion into the cell. Background fluorescence intensity is subtracted in each plot. Initial fluorescence of the nanoneedle is represented as relative fluorescence intensity of ‘1’. n = 4 for each condition. Two-sided Student’s *t*-test was performed on the data at the time point of 10 min. *P = 0.019, **P = 0.0014. Error bars are SD. Scale bars in (**a**) are 10 μm.

To confirm successful insertion of the nanoneedles into the cells, we used a molecular beacon (MB) which was designed to fluoresce upon hybridization with human GAPDH mRNA^22^. MBs were immobilized on the nanoneedles through biotynilation reaction between the biotin-modified MBs and streptavidin that was bound to BSA, which was adsorbed on the nanoneedle surface^25^. Supplementary Fig. 2 shows results for the *in vitro* assay, demonstrating increase in the relative fluorescence following addition of 1 μM ssDNA. For confirming insertion into live cells, the MB-functionalized nanoneedle array was lowered onto DsRed2-NES-expressing HEK293 cells^16^ and was imaged using confocal laser scanning microscope (CLSM). Figure 3a demonstrates successful penetration of the nanoneedles through the cell membrane. The fluorescence intensities of the nanoneedles gradually increased following insertion within 10 min, in accord with binding of intracellular mRNA to the immobilized MBs on the nanoneedles (Fig. 3b). The stronger intensity observed in the cytosol compared to that in the nucleus reflects GAPDH mRNA localization in the cytoplasm of cells. The 30-μm-pitch nanoneedle array also showed MB response (Supplementary Fig. 3). These reproducible results show direct insertion of the nanoneedles through the membrane and into the cytosol with high efficacy. Moreover, as a negative control, we inserted MB-immobilized nanoneedles into mouse NIH3T3 cells, which has three mismatches in their GAPDH mRNA, compared to human HEK293 cells. As the MBs used were specific for human GAPDH mRNA, no significant fluorescence increase was observed following insertion into the mouse cells (Supplementary Fig. 4).

### Delivery of plasmid DNA into live cells

**Figure 4:**
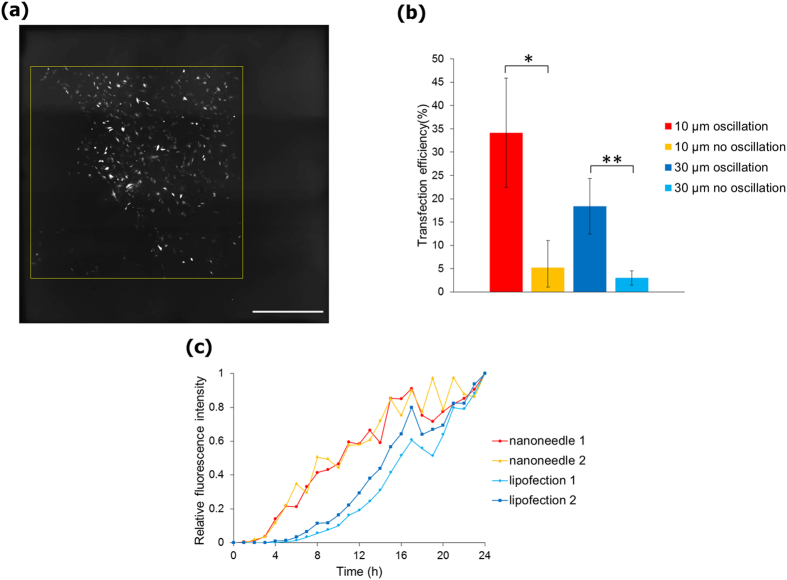
Delivery of plasmid DNA with the use of the nanoneedle array. (**a**) Fluorescent images of NIH3T3 cells transfected with pCS2-Venus plasmids using a 10 μm-pitch nanoneedle array. Yellow square of 3 × 3 mm represents the contact area covered with the nanoneedle array. (**b**) Transfection efficiencies using the nanoneedle array with and without oscillation. n = 3 for each condition. Two-sided Student’s *t*-test was performed. *P = 0.036, **P = 0.024. Error bars are SD. (**c**) Time course of relative fluorescence intensity after introduction of pCS2-Venus. Cells transfected with plasmid DNAs were observed by fluorescence microscopy and fluorescence intensities were analyzed from images with use of ImageJ. Fluorescence intensity after 24 h is indicated as 1. Scale bar in (**a**) is 10 μm.

Plasmid DNA encoding the fluorescent protein Venus was adsorbed on the surface of the nanoneedle array. The nanoneedle array was then lowered down and nanoneedles were inserted into NIH3T3 cells. Following insertion, the chip was oscillated using the piezo pantograph with amplitude of 1.0 μm at a frequency of about 5 kHz (Supplementary Fig. 5). After 24 h incubation, cells expressing the Venus fluorescent protein were only observed in the area where the nanoneedle array contacted (Fig. 4a). The highest efficiency of plasmid expression reached 34% (percentage of cells transfected), in the case of nanoneedle array with 10 μm pitch and with oscillation. The 30 μm pitch nanoneedle array resulted in lower transfection efficiency, due to the lower density of nanoneedles, which led to fewer cells being inserted. When a flat silicon plate without nanoneedles was used, the efficiency of transfection was only 0.7%. These results demonstrate the use of the nanoneedle array for a rapid and efficient delivery of naked plasmid DNA into live cells. In addition, the use of oscillation had an obvious contribution to the efficiency of plasmid transfer, showing an increase of 7-fold and 6-fold when using oscillatory mode compared to non-oscillatory mode, for 10 μm and 30 μm pitch, respectively, as can be seen in Fig. 4b. This result shows that the piezo pantograph system has a significant contribution to the release of adsorbed molecules from the silicon surface. Moreover, the expression of fluorescent proteins in cells transfected using the nanoneedle array were more rapid than in the case of lipofection transfection, as measured by the relative fluorescent intensity over time (Fig. 4c), possibly due to direct delivery of plasmid DNA into the nucleus.

### Delivery of Cre recombinase

**Figure 5:**
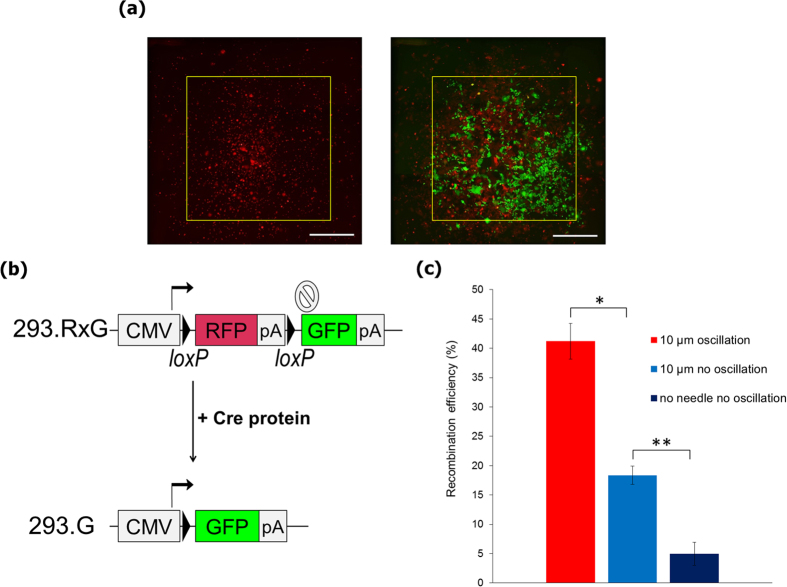
Delivery of Cre recombinase into live cells using the nanoneedle array. (**a**) Fluorescent images of 293.RxG cells transfected with the Cre protein with use of 10 μm pitch nanoneedle array after 1 min (left) and 48 h (right). Following successful insertion, cells that underwent recombination due to Cre delivery express GFP instead of RFP, showing in green color. Yellow square of 3 × 3 mm represents the contact area covered with the nanoneedle array. Scale bars are 1 mm. (**b**) Structure of the reporter gene in 293.RxG cells before and after recombination with Cre protein. (**c**) Recombination efficiency with Cre protein delivered by 10 μm pitch nanoneedle array with or without oscillation and with use of a flat silicon wafer (n = 3). Two-sided Student’s *t*-test was performed. *P = 0.0026, **P = 0.0021. Error bars are SD.

To further test the versatility of the nanoneedle array system, we used it for the delivery of Cre recombinase, as a representative functional protein that mediates genomic DNA recombination. We first established a reporter cell line, 293.RxG, by stably integrating an expression cassette encoding the gene for red fluorescence protein (RFP) followed by a transcription termination signal and a sequence encoding for green fluorescence protein (GFP) into the genome of HEK293 cell line. After two *loxP* sites are excised, with the introduction of Cre recombinase, the 293.RxG cells, originally expressing RFP, change their color from red to green by expressing GFP (Fig. 5b). Purified Cre proteins were then adsorbed on the nanoneedle array by incubating at RT for 60 min, and were delivered by insertion into the 293.RxG cells. The delivered Cre proteins spontaneously translocated into the nucleus due to nuclear localization signal fused to their N-terminus. After 48 h of incubation, many GFP-positive cells could be observed, demonstrating efficient transfer of Cre proteins (Fig. 5a). Small number of cells expressing both RFP and GFP can also be observed, as some non-degraded RFP remained even after Cre recombination. The effect of oscillation was evident here, as in the case of plasmid DNA delivery, suggesting an increase in protein release due to the oscillation of the array (Fig. 5c).

## Discussion

The number of GAPDH mRNAs in a single MCF-7 cell, approximately 2600, was reported in previous work^26^. Similar numbers of mRNAs were determined for single HeLa and CHO-K1 cells by Dr. Takafumi Mizuno of AIST (personal communication). We can assume similar levels of GAPDH mRNAs transcription in HEK293 cells. Considering the number of GAPDH mRNAs, the fluorescence intensities are relatively high. However, the quantum efficiency of the beacon-immobilized nanoneedle in the cytosolic environment is not known. Therefore, we do not analyze here the kinetics of mRNAs association with MBs but rather evaluate the relative increase of fluorescence intensities as a proof of successful nanoneedle insertion into the cells.

The method presented here, using high-aspect-ratio nanoneedles coupled with an oscillation system, allowed for a high efficiency transfer of biomolecules into the live cells by facilitating the release of needle-adsorbed molecules. In particular, the efficiency of naked plasmid DNA delivery presented here was improved to a great extent over previous reports that used force-loading methods. Further improvements in the efficiency of nanoneedles insertion into the cells, more effective anchoring of biomolecules on the silicon surface of the nanoneedle, and more accurate control of biomolecule release inside the cell or nucleus, will increase the potential of this system for use as an efficient way for large-scale, high-throughput delivery system.

In this work, we have established a simple protocol for the processing of a silicon wafer, which consists of photolithography, ICP dry etching and removal of deep layer of SiO_2_, resulting in the fabrication of a nanoneedle array containing several tens of thousands of nanoneedles with an aspect ratio of over one hundred, evenly spread in an area of 3 mm^2^. A specially-designed nanoneedle-array manipulator was combined with the system to allow approaching the array chip to facilitate forcible, simultaneous insertion of the nanoneedles into live cells cultured on petri dishes. In addition, the manipulator’s unique vertical oscillation function enables rapid biomolecule delivery with high efficiencies. In as little as one-minute insertion, naked plasmid DNA was successfully transferred into NIH3T3 cells with efficiency of 34% and Cre recombinase was delivered with an efficiency of 42%. The developed nanoneedle array chip and manipulator could be further utilized as a versatile tool to manipulate living cells. Recently, site-specific nucleases, such as the CRISPR/Cas9 system, were delivered as ribonucleoproteins in order to reduce off-target effects in genome editing^27^,^28^,^29^. Our system can easily be employed for delivery of such functional molecules. In addition, thanks to its high throughput, this system can be further developed and applied as novel cell sorting technology for medical and clinical applications.

## Methods

### Nanoneedle array manipulation

The nanoneedle array manipulator was placed on the stage of an inverted microscope (GX71, Olympus). A nanoneedle array chip was attached to the array holder (aluminum cube) with double-sided adhesive tape, and horizontal aligning of the array chip was performed by using a high N.A. lens (MPLFLN150xBD, Olympus) and making sure the nanoneedles’ tips at the four corners of the array are all in the same focal plane. DNA or protein was adsorbed to the silicon surface of the chip. The nanoneedle array was first positioned approximately 200 μm above the cells, and was then approached towards the cells at a speed of 2.5 μm/s by using the Z piezo motor, while observing to confirm that the nanoneedles come into contact with the dish. Then, the array was retracted back several μm. The chip was then held still, or oscillated at about 5 kHz using the piezo pantograph and the function generator, for 1 min, to allow release of the adsorbed molecules. Following that, the chip was retracted and the nanoneedles were evacuated from the cells, at the same speed as that in the approach.

### *In vitro* assay for measuring the response of molecular beacon to target sequences

We made cytosol-mimicking buffer (20 mM HEPES, 115 mM CH_3_COOK, 5 mM MgCl_2_, 1 mM DTT, pH 7.1). Fluorescence imaging was carried out using CLSM (IX71/FV300, Olympus). We pipetted 200 μl of the cytosol-mimicking buffer on 12mmϕ glass-base dish (IWAKI). Molecular beacon (MB) was purchased from Medical & Biological Laboratories. The MB was labeled with Alexa 488 at the 5′ end and with biotin and black hole quencher (BHQ-1) at the 3′ end (5′-Alexa488-ACGACGGAGTCCTTCCACGATACCACGTCGbiotinT-BHQ1-3′). We modified nanoneedle arrays with MBs using biotinylated BSA (Sigma), streptavidin (Wako), and biotinylated MBs. First, we cleaned the surface of the nanoneedle array using SPM solution at 80 °C for 10 min. We then rinsed with ultrapure water (Kanto Chemical), and immersed the nanoneedle array in 1% HF solution for 1 min in order to make the surface hydrophobic by removing SiO_2_ layer, followed by washing with ultrapure water. Then, the nanoneedle array was incubated at RT for 60 min in 1.5 μM biotinylated BSA solution, and then washed twice with PBS. The biotinylated-BSA-adsorbed nanoneedle array was then immersed in 1.5 μM streptavidin solution and incubated at RT for 60 min, followed by washing twice with PBS. Finally, the streptavidin-modified array was incubated in 1.5 μM MB solution at RT for 60 min, and then washed twice with PBS. We fixed the MB-modified-nanoneedle array on the array holder. Then, we lowered the nanoneedle array to the buffer by manual Z-axis adjustment, until the nanoneedle array was near the base of the dish. We focused on the tips of the nanoneedles, and scanned from the tip upwards 30 μm at a scan rate of 1 μm/slice (total 31 slices). Then, we added 2 μl of 100 μM oligo DNA which had human GAPDH sequence (final concentration of 1 μM, Life Technologies). We repeated the scan again and analyzed the change in fluorescence intensity. In the analysis we took into consideration the fluorescence intensity of the nanoneedles from the tip to the 10 μm upper part using the image analyzing software ImageJ.

### *In vivo* assay for measuring the response of molecular beacon to target sequences

HEK293 expressing DsRed2-NES (10^5^ cells) were seeded on μ-dish (35 mm, high, ibidi) and incubated at 37 °C for 24 h in growth media (GM, DMEM (Sigma), to which were added 10% FBS (Gibco), 2 mM Glutamax (Life Technologies), 4 μl of gentamycin/amphotericin (Life Technologies)). DsRed2-NES was used in order to confirm nucleus position. The medium was exchanged to Opti-MEM (Life Technologies) before the insertion test. The MB-modified nanoneedle array (the method of modification is described prior section) was then approached to the cells using the manipulator until contact with surface of the dish was confirmed. Then, the array was retracted back several μm and was held still (no oscillation). Confocal fluorescence images of the nanoneedles and cells were acquired starting from the base of the culture dish up to a height of 30 μm, every 1 μm. Fluorescence intensity of the nanoneedles was analyzed taking into account the first 10-μm segment of the nanoneedle, from the tip towards the base, using ImageJ.

### Measurement of the array manipulator’s resonance frequency

At high frequencies in the kHz range, the oscillation amplitude of the chip mounted on the holder cannot follow the actual amplitude of the piezoelectric actuator (piezo pantograph). The vibration of the entire apparatus determines the oscillation characteristic of the chip. Thus, it is required to measure the resonance frequency of the chip and chose an appropriate frequency that shows amplitude that is as close as possible to the drive amplitude. The nanoneedle array chip was oscillated using the piezo pantograph. To determine the oscillation frequency of the needle, the frequency response of the piezoelectric actuator was obtained using a network analyzer by applying a range of frequencies to the piezoelectric actuator. The amplitude of the piezoelectric actuator was detected using a fiber-optic displacement sensor (Philtec, Inc.). From the resonance peaks of the frequency response of the actuator, harmonic frequency of around 5 kHz was used to oscillate the nanoneedles.

### Delivery of plasmid DNA into the cell using the nanoneedle array

pCS2-Venus plasmid was used in the delivery test. Cleaning procedure and HF treatment of the nanoneedle array prior to plasmid DNA adsorption was same as previously described. The nanoneedle array was immersed with 12 μl of 67% ethanol solution containing 2 μg of pCS2-Venus plasmid and 0.6 μmol sodium acetate, and was incubated at −20 °C for 30 min. Then, the DNA solution was dried by incubating at RT for 30–60 min. NIH3T3 (10^4^ cells in 10 μl) were seeded on a center of a collagen-coated culture dish (40 mm, TPP) and incubated at 37 °C for 30 min. Then, 2 ml of GM was added to the dish and it was incubated for 3 h, prior to DNA delivery test. Following insertion of the nanoneedles into the cells, the cells were rinsed with PBS to remove plasmid remaining in the medium. Fresh GM was then added and the cells were incubated for 24 h before being subjected to fluorescence imaging. We calculated delivery efficiency from the area of Venus-expressing cells relative to the total area of cells as seen in the bright field image, using Image-Pro Plus software (Media Cybernetics).

### Delivery of Cre recombinase using the nanoneedle array

Cre proteins were expressed using *E. coli* BL21(DE3) strain carrying the plasmid pETHNCre. *E. coli* was cultured at 25 °C for 6 h following 0.1 mM IPTG induction. Cre proteins were purified using Ni-NTA column with 20 mM imidazole wash and 500 mM imidazole elution buffer containing TBSG (10 mM Tris-HCl, 500 mM NaCl, 10% glycerol, pH 8.0). Proteins were dialyzed against HBSG (20 mM HEPES, 150 mM NaCl, 10% glycerol, pH 7.4). A hydrophobized nanoneedle array was immersed with 10 μl of 100 μM Cre protein solution and incubated at RT for 60 min. The array chip was then rinsed with HBS buffer before fixing on the array holder. We prepared 293.RxG cells by the following method: A plasmid pcDNAFRTxE2CxmEm, which encodes GFP (mEmerald) and RFP (E2-Crimson) sandwiched by two *loxP* sites, was stably integrated into Flp-In-293 cells (Life Technologies) according to the manufacturer’s protocol. A clone of 293.RxG was selected with hygromycin, which expresses RFP at normal conditions. The method for culturing 293.RxG cells and insertion of the nanoneedles into cells, were same as described in the previous section. We observed fluorescence change 48 h after treatment. We calculated the efficiency of delivery from the area of GFP-expressing cells and RFP-expressing cells using Image-Pro Plus.

## Additional Information

**How to cite this article**: Matsumoto, D. *et al.* Oscillating high-aspect-ratio monolithic silicon nanoneedle array enables efficient delivery of functional bio-macromolecules into living cells. *Sci. Rep.* **5**, 15325; doi: 10.1038/srep15325 (2015).

## Supplementary Material

Supplementary Information

## References

[b1] Inoue H., Nagata N., Kurokawa H. & Yamanaka S. iPS cells: a game changer for future medicine. EMBO J 33, 409–417 (2014). 24500035 10.1002/embj.201387098 PMC3989624

[b2] Mocellin S. & Provenzano M. RNA interference: learning gene knock-down from cell physiology. J Transl Med 2, 39 (2004). 15555080 10.1186/1479-5876-2-39 PMC534783

[b3] Gaj T., Gersbach C. A. & Barbas C. F. 3rd ZFN, TALEN, and CRISPR/Cas-based methods for genome engineering. Trends Biotechnol 31, 397–405 (2013). 23664777 10.1016/j.tibtech.2013.04.004 PMC3694601

[b4] Reissmann S. Cell penetration: scope and limitations by the application of cell-penetrating peptides. J Pept Sci 20, 760–784 (2014). 25112216 10.1002/psc.2672

[b5] Kay M. A., Glorioso J. C. & Naldini L. Viral vectors for gene therapy: the art of turning infectious agents into vehicles of therapeutics. Nat Med 7, 33–40 (2001). 11135613 10.1038/83324

[b6] Dalby B. *et al.* Advanced transfection with Lipofectamine 2000 reagent: primary neurons, siRNA, and high-throughput applications. Methods 33, 95–103 (2004). 15121163 10.1016/j.ymeth.2003.11.023

[b7] De Smedt S. C., Demeester J. & Hennink W. E. Cationic polymer based gene delivery systems. Pharm Res 17, 113–126 (2000). 10751024 10.1023/a:1007548826495

[b8] Varkouhi A. K., Scholte M., Storm G. & Haisma H. J. Endosomal escape pathways for delivery of biologicals. J Control Release 151, 220–228 (2011). 21078351 10.1016/j.jconrel.2010.11.004

[b9] Mellott A. J., Forrest M. L. & Detamore M. S. Physical non-viral gene delivery methods for tissue engineering. Ann Biomed Eng 41, 446–468 (2013). 23099792 10.1007/s10439-012-0678-1 PMC5102682

[b10] Xie C., Lin Z., Hanson L., Cui Y. & Cui B. Intracellular recording of action potentials by nanopillar electroporation. Nat Nanotechnol 7, 185–190 (2012). 22327876 10.1038/nnano.2012.8 PMC3356686

[b11] Na Y. R. *et al.* Probing enzymatic activity inside living cells using a nanowire-cell “sandwich” assay. Nano Lett 13, 153–158 (2013). 23244056 10.1021/nl3037068 PMC3541459

[b12] Shalek A. K. *et al.* Nanowire-mediated delivery enables functional interrogation of primary immune cells: application to the analysis of chronic lymphocytic leukemia. Nano Lett 12, 6498–6504 (2012). 23190424 10.1021/nl3042917 PMC3573729

[b13] Xu A. M. *et al.* Quantification of nanowire penetration into living cells. Nat Commun 5, 3613 (2014). 24710350 10.1038/ncomms4613 PMC6057472

[b14] Peer E., Artzy-Schnirman A., Gepstein L. & Sivan U. Hollow nanoneedle array and its utilization for repeated administration of biomolecules to the same cells. ACS Nano 6, 4940–4946 (2012). 22632128 10.1021/nn300443h

[b15] Berthing T. *et al.* Cell membrane conformation at vertical nanowire array interface revealed by fluorescence imaging. Nanotechnology 23, 415102 (2012). 23010859 10.1088/0957-4484/23/41/415102

[b16] Obataya I., Nakamura C., Han S., Nakamura N. & Miyake J. Nanoscale operation of a living cell using an atomic force microscope with a nanoneedle. Nano Lett 5, 27–30 (2005). 15792407 10.1021/nl0485399

[b17] Angle M. R., Wang A., Thomas A., Schaefer A. T. & Melosh N. A. Penetration of cell membranes and synthetic lipid bilayers by nanoprobes. Biophys J 107, 2091–2100 (2014). 25418094 10.1016/j.bpj.2014.09.023 PMC4223211

[b18] McKnight T. E. *et al.* Intracellular integration of synthetic nanostructures with viable cells for controlled biochemical manipulation. Nanotechnology 14, 551–556 (2003).

[b19] Wang Y. *et al.* Poking cells for efficient vector-free intracellular delivery. Nat Commun 5, 4466 (2014). 25072981 10.1038/ncomms5466

[b20] Han S. W. *et al.* High-efficiency DNA injection into a single human mesenchymal stem cell using a nanoneedle and atomic force microscopy. Nanomedicine 4, 215–225 (2008). 18501680 10.1016/j.nano.2008.03.005

[b21] Han S. W., Nakamura C., Obataya I., Nakamura N. & Miyake J. A molecular delivery system by using AFM and nanoneedle. Biosens Bioelectron 20, 2120–2125 (2005). 15741084 10.1016/j.bios.2004.08.023

[b22] Kihara T. *et al.* Development of a novel method to detect intrinsic mRNA in a living cell by using a molecular beacon-immobilized nanoneedle. Biosens Bioelectron 26, 1449–1454 (2010). 20708917 10.1016/j.bios.2010.07.079

[b23] Mieda S. *et al.* Mechanical force-based probing of intracellular proteins from living cells using antibody-immobilized nanoneedles. Biosens Bioelectron 31, 323–329 (2012). 22093769 10.1016/j.bios.2011.10.039

[b24] Han S., Nakamura C., Obataya I., Nakamura N. & Miyake J. Gene expression using an ultrathin needle enabling accurate displacement and low invasiveness. Biochem Biophys Res Commun 332, 633–639 (2005). 15925564 10.1016/j.bbrc.2005.04.059

[b25] Rostgaard K. R. *et al.* Vertical nanowire arrays as a versatile platform for protein detection and analysis. Nanoscale 5, 10226–10235 (2013). 24062006 10.1039/c3nr03113f

[b26] Nashimoto Y. *et al.* Measurement of gene expression from single adherent cells and spheroids collected using fast electrical lysis. Anal Chem 79, 6823–6830 (2007). 17676760 10.1021/ac071050q

[b27] Kim S., Kim D., Cho S. W., Kim J. & Kim J. S. Highly efficient RNA-guided genome editing in human cells via delivery of purified Cas9 ribonucleoproteins. Genome Res 24, 1012–1019 (2014). 24696461 10.1101/gr.171322.113 PMC4032847

[b28] Ramakrishna S. *et al.* Gene disruption by cell-penetrating peptide-mediated delivery of Cas9 protein and guide RNA. Genome Res 24, 1020–1027 (2014). 24696462 10.1101/gr.171264.113 PMC4032848

[b29] Zuris J. A. *et al.* Cationic lipid-mediated delivery of proteins enables efficient protein-based genome editing *in vitro* and *in vivo*. Nat Biotechnol 33, 73–80 (2015). 25357182 10.1038/nbt.3081 PMC4289409

