# Supplementary material for: Oscillating high-aspect-ratio monolithic silicon nanoneedle array enables efficient delivery of functional bio-macromolecules into living cells
Source: Sci Rep. 2015 Oct 16;5:15325. doi: 10.1038/srep15325 (PMC4607922; doi:10.1038/srep15325)
Supplement: Supplementary Information [file srep15325-s1.pdf]

Oscillating high-aspect-ratio monolithic silicon nanoneedle array enables efficient delivery of functional bio-macromolecules into living cells

Daisuke Matsumoto[1,2], Ramachandra Rao Sathuluri[2], Yoshio Kato[2], Yaron R. Silberberg[2], Ryuzo Kawamura[2], Futoshi Iwata[3], Takeshi Kobayashi[4] and Chikashi Nakamura[1,2]

[1]Department of Biotechnology and Life Science, Tokyo University of Agriculture and Technology, 2-24-16 Naka-cho, Koganei, Tokyo, 184-8588, Japan
[2]Biomedical Research Institute, National Institute of Advanced Industrial Science and Technology (AIST), Central4 1-1-1 Higashi, Tsukuba, Ibaraki, 305-8562, Japan
[3]Department of Mechanical Engineering, Shizuoka University, 3-5-1 Johoku, Hamamatsu 432-8561, Japan
[4]Research Center for Ubiquitous MEMS and Micro Engineering, AIST, 1-2-1 Namiki, Tsukuba, Ibaraki 305-8564, Japan

Supplementary Figures

a

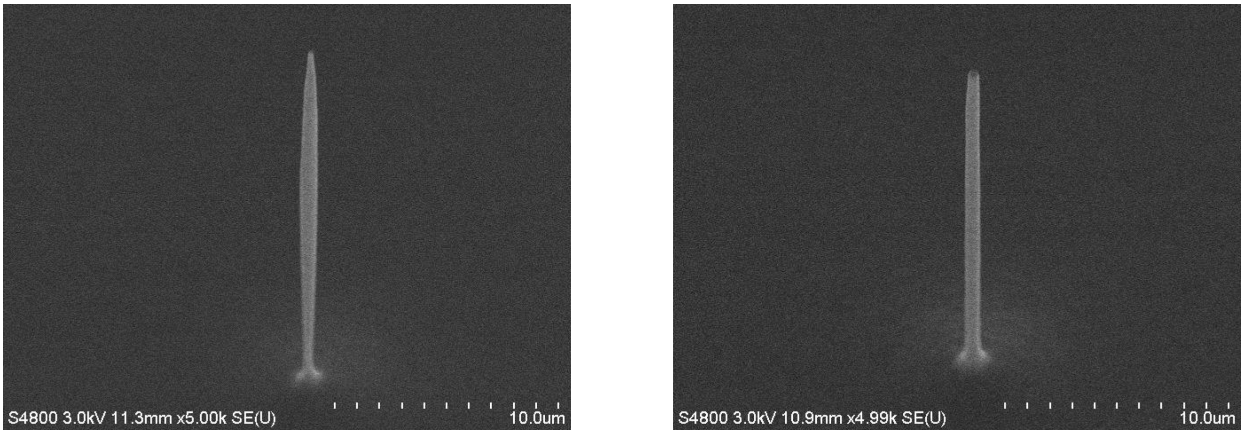

b

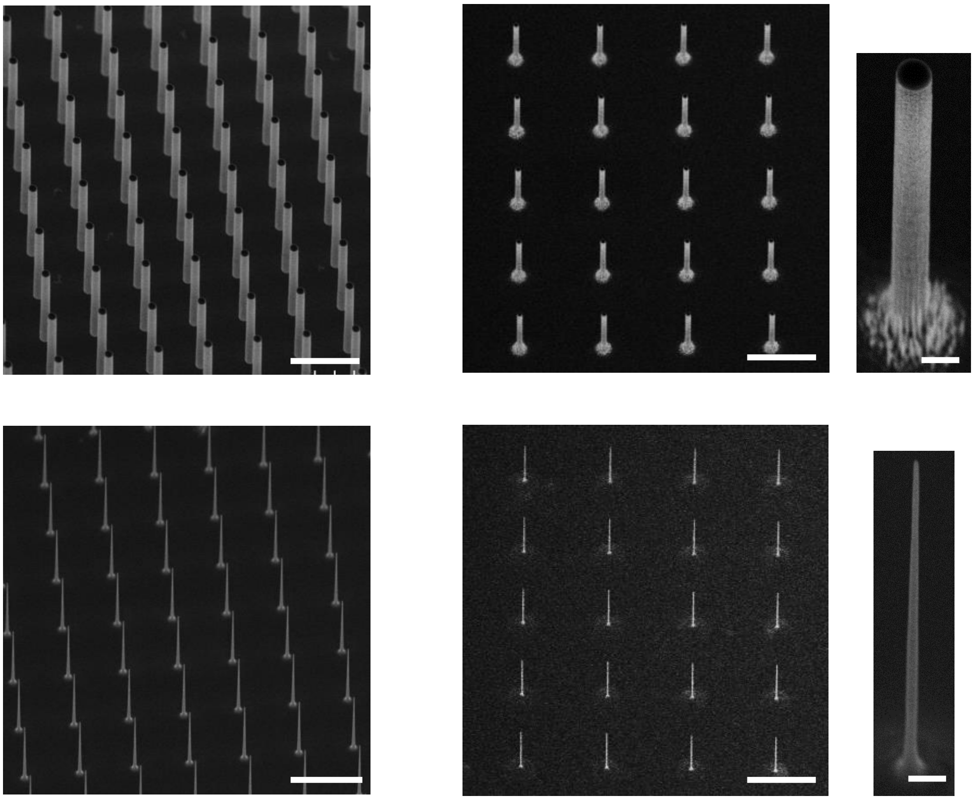

Supplementary Fig. 1 | a) FE-SEM images of nanoneedles. A constant etching time often resulted in nanoneedles with overly-narrow bottom part (left). Gradually decreasing the etching time resulted in a more uniform nanoneedle (right). b) FE-SEM images of fabricated micropillar and nanoneedle arrays. Micropillar array (top) and nanoneedle array (bottom) are spaced out uniformly. Scale bars in nanoneedle- and micropillar-array images are 20 μm, and those in the single nanoneedle and micropillar images are 2 μm. These samples are tilted 30°.

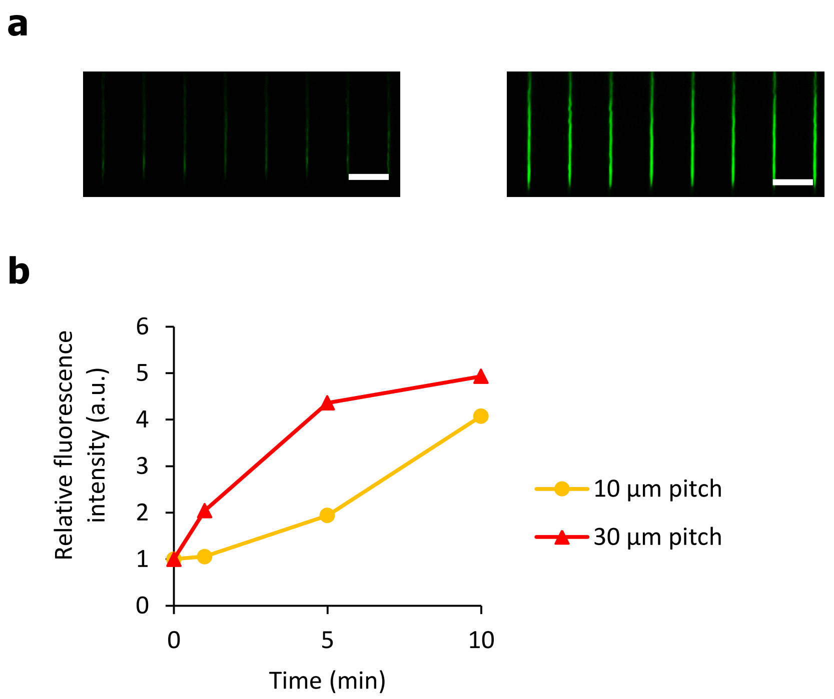

Supplementary Fig. 2 | *In vitro* assay of molecular beacons immobilized on the nanoneedles' surface. a) XZ images of 10 µm pitch molecular beacon-immobilized nanoneedle array, reconstructed from a stack of CLSM images before (left) and 10 min after (right) addition of target single strand DNA (5'-AACTTTGGTATCTTTGGTATCGTGGAAGGACTCATGACG). b) Time course of the relative fluorescent intensities of the nanoneedles after addition of 1 µM ssDNA. The relative fluorescence prior to target sequence addition is set as '1'. Scale bars in a are 10 µm.

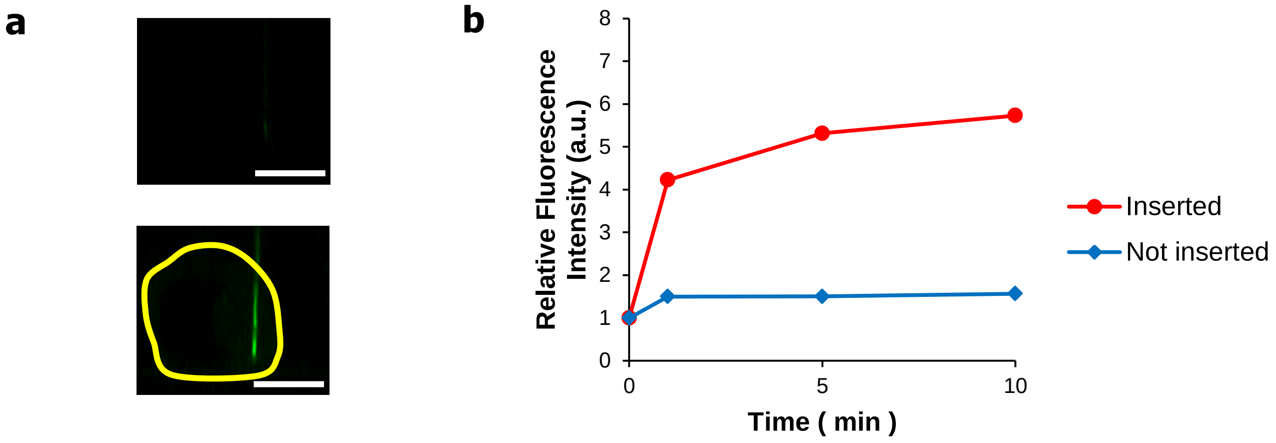

Supplementary Fig. 3 | Response of molecular beacon *in vivo* using 30 µm pitch nanoneedle array. a) YZ CLSM images of MB-modified nanoneedle inserted into cytosol of HEK293 cell. Top image was scanned right after insertion, and bottom image was scanned 10 min after insertion. Yellow circle shows the outline of the cell. b) Time courses of relative fluorescence intensities of the nanoneedles in the cytosol following insertion. Time-point '0' indicates prior of nanoneedle insertion. Relative fluorescence intensities are determined relatively to time-point '0'. Scale bars in a are 10 µm.

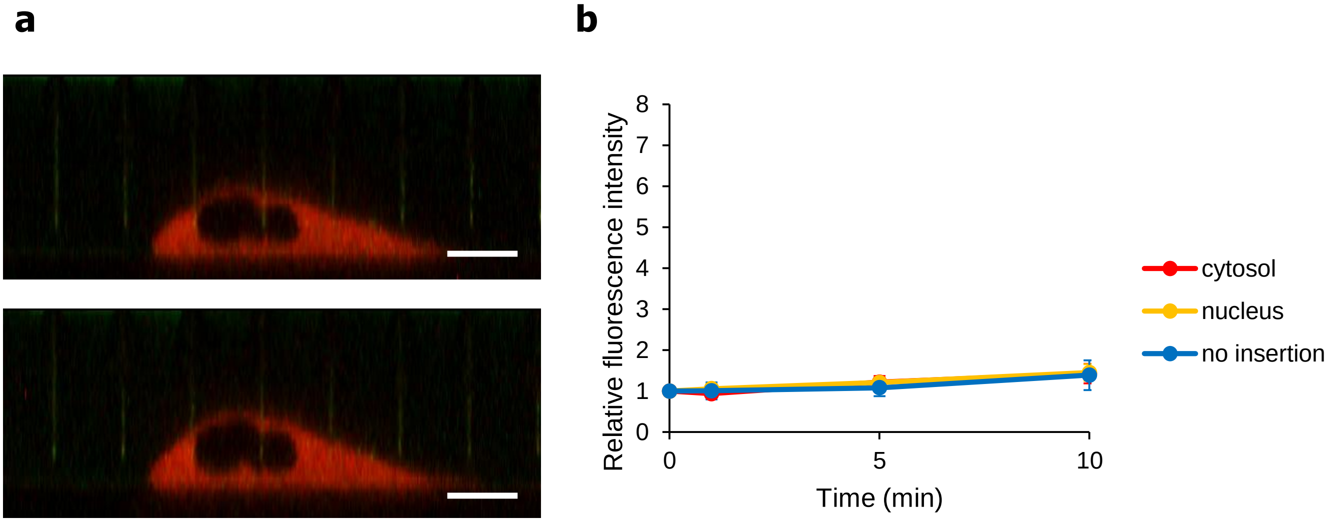

Supplementary Fig. 4 | Lack of fluorescence response for the human-specific molecular beacon when inserted into mouse NIH3T3 cells. a) YZ CLSM images of MB-modified nanoneedle inserted into cytosol and nucleus of NIH3T3 cell 1 min (top) and 10 min (bottom) after insertion. b) Time courses of relative fluorescence intensities of the nanoneedles in the cytosol, nucleus, and medium following insertion. Time-point ‘0’ indicates prior for nanoneedle insertion. Relative fluorescence intensities are determined relatively to time-point ‘0’. n=4 for each condition. Error bars are SD. Scale bars in a are 10 µm.

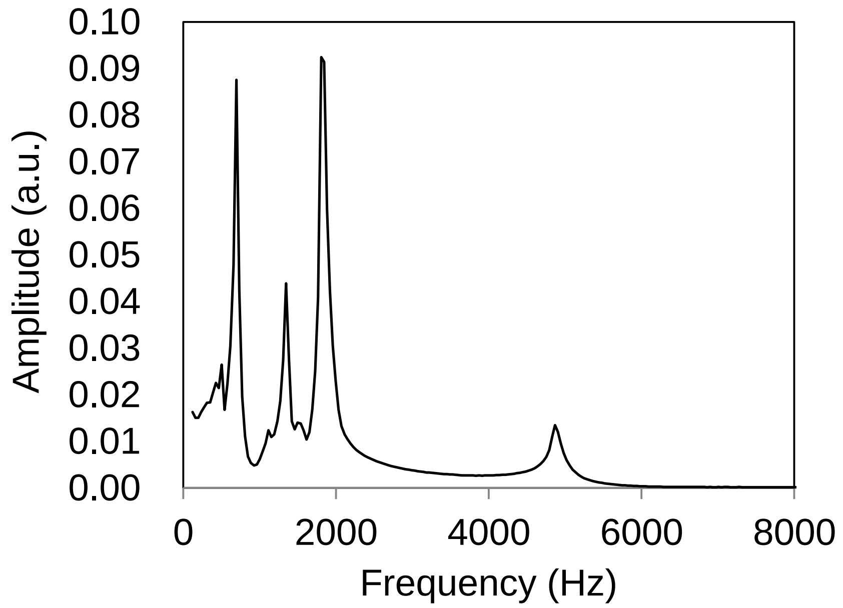

Supplementary Fig. 5 | Oscillation resonance frequency spectrum of the array holder of the manipulator. The harmonic frequencies are detected at about 700 Hz, 1400 Hz, 1700 Hz, 5000 Hz.

Supplementary Tables

Supplementary table 1 | Averaged diameters and lengths of micropillar arrays in chips

	10 µm pitch micropillar array					30 µm pitch micropillar array				
	Chip1	Chip2	Chip3	Chip4	Chip5	Chip 1	Chip2	Chip3	Chip4	Chip5
Diameter (µm)	1.71	1.54	1.90	1.92	1.90	1.88	1.97	1.96	1.98	1.99
SD	0.09	0.08	0.04	0.04	0.06	0.04	0.04	0.05	0.04	0.05
CV (%)	5.08	4.94	2.23	2.15	2.91	1.87	1.78	2.43	2.24	2.31
Length (µm)	23.6	24.3	24.7	22.5	22.4	24.4	24.3	24.4	24.6	24.6
SD	0.87	0.71	0.27	1.14	0.49	0.57	0.43	0.28	0.30	0.35
CV (%)	3.68	2.92	1.10	5.07	2.19	2.32	1.79	1.16	1.20	1.42

Supplementary table 2 | Averaged diameters and lengths of nanoneedle arrays in chips

	10 µm pitch nanoneedle array					30 µm pitch nanoneedle array				
	Chip1	Chip2	Chip3	Chip4	Chip5	Chip 1	Chip2	Chip3	Chip4	Chip5
Diameter (nm)	220	194	278	171	174	227	287	311	269	192
SD	22.6	37.5	26.2	37.7	41.3	27.0	19.5	39.9	36.3	27.5
CV (%)	10.3	19.3	9.4	22.1	23.7	11.9	6.8	12.9	13.5	14.4
Length (µm)	27.1	27.0	30.1	27.0	26.3	24.9	24.9	24.5	24.0	23.5
SD	0.42	0.33	0.35	0.57	0.84	0.26	0.41	0.25	0.26	1.22
CV (%)	1.57	1.22	1.16	2.12	3.20	1.05	1.66	1.02	1.06	5.20
